# Case Report: Fabry disease presenting with electrocardiographic findings mimicking acute myocardial infarction: a diagnostic challenge

**DOI:** 10.3389/fcvm.2026.1727546

**Published:** 2026-02-03

**Authors:** Jin-Mei Xie, Qing Li, Zhi-Qiang Xiao, Zong-Jie Zheng

**Affiliations:** 1 Departments of Electrocardiographic Diagnosis, Cardiovascular Medicine, and Ultrasound Medicine, Sanming First Affiliated Hospital to Fujian Medical University, Sanming, Fujian, China; 2 Department of Electrocardiogram, The Second Affiliated Hospital, School of Medicine, Zhejiang University, Hangzhou, China; 3 State Key Laboratory of Transvascular Implantation Devices, Hangzhou, China; 4 Heart Regeneration and Repair Key Laboratory of Zhejiang Province, Hangzhou, China

**Keywords:** case report, electrocardiography, Fabry disease, genetic testing, left ventricular hypertrophy, pseudo-infarction

## Abstract

Fabry disease (FD) is a rare X-linked lysosomal storage disorder characterized by heterogeneous clinical manifestations. Cardiac involvement arises from progressive glycosphingolipid accumulation within cardiomyocytes, vascular endothelium, and the conduction system, resulting in left ventricular hypertrophy, conduction abnormalities, and arrhythmias. A subset of patients exhibits electrocardiographic findings that mimic acute myocardial infarction (AMI), often leading to misdiagnosis as ischemic heart disease.

**Case presentation:**

We describe a 62-year-old man presenting with recurrent chest pain and syncope. Initial electrocardiography demonstrated pathological Q waves and ST-segment elevation in the anterior leads. Echocardiography revealed concentric left ventricular hypertrophy. Serial cardiac biomarkers, repeat echocardiography, and coronary angiography excluded AMI. The diagnosis of FD was confirmed by markedly reduced leukocyte α-galactosidase A activity, elevated plasma lyso-Gb3 concentrations, and identification of a pathogenic hemizygous variant in the GLA gene.

**Conclusion:**

In patients with unexplained left ventricular hypertrophy and “pseudo-infarction” electrocardiographic patterns, targeted evaluation for FD is warranted to prevent diagnostic delay and enable timely initiation of enzyme replacement therapy (ERT) and multidisciplinary management.

## 1 Introduction

Fabry disease is an X-linked recessive lysosomal storage disorder caused by pathogenic variants in the GLA gene, which encodes the enzyme α-galactosidase A. Deficiency of this enzyme results in progressive intracellular accumulation of globotriaosylceramide and related glycosphingolipids across multiple organ systems. Cardiac involvement is common and manifests as concentric left ventricular hypertrophy, conduction abnormalities, and repolarization disturbances (1). A distinct clinical phenotype—cardiac-variant Fabry disease—presents in adulthood with isolated cardiac manifestations, lacking the classic dermatologic, renal, or neurologic features that typically facilitate early diagnosis (2). Consequently, these patients are frequently misdiagnosed with more prevalent cardiovascular conditions.

## 2 Case presentation

A 62-year-old man presented to the Sanming First Affiliated Hospital of Fujian Medical University on May 31, 2025, with recurrent chest pain and a syncopal episode. In the preceding 48 h, he experienced multiple episodes of retrosternal, crushing chest pain, each lasting approximately 30 min, without radiation, and resolving spontaneously. While ambulating at 3:00 p.m. on the day of presentation, he abruptly lost consciousness. Witnesses reported a fall lasting approximately one minute, with spontaneous recovery; the patient had no memory of the event. His medical history was negative for hypertension and diabetes. Family history was significant for sudden cardiac death in his mother at age 50, who was reportedly diagnosed with hypertrophic cardiomyopathy, although detailed medical records were unavailable.

**Figure 1:**
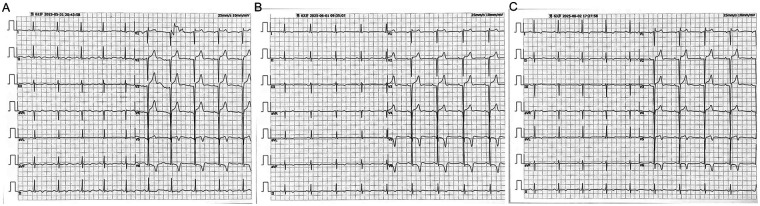
Twelve-lead electrocardiograms. **(A)** Shows the tracing obtained at admission. **(B,C)** Depict recordings from hospital days 2 and 3, respectively, demonstrating no dynamic ST-T segment changes.

Admission electrocardiography (Figure 1A) demonstrated sinus rhythm with incomplete right bundle-branch block, pathological Q waves (>40 ms duration and amplitude ≥25% of the subsequent R wave) in leads V2 through V4, and 2–3 mm upsloping ST-segment elevation in the same leads. T-wave flattening or inversion was noted in leads I, II, aVL, aVF, V5, and V6. Serial electrocardiograms on hospital days 2 and 3 (Figures 1B,C) showed no dynamic ST-T changes.

Laboratory evaluation revealed normal renal function, absence of proteinuria, and serum levels of creatine kinase, CK-MB, cardiac troponin I, myoglobin, and N-terminal pro–B-type natriuretic peptide within reference ranges.

**Figure 2:**
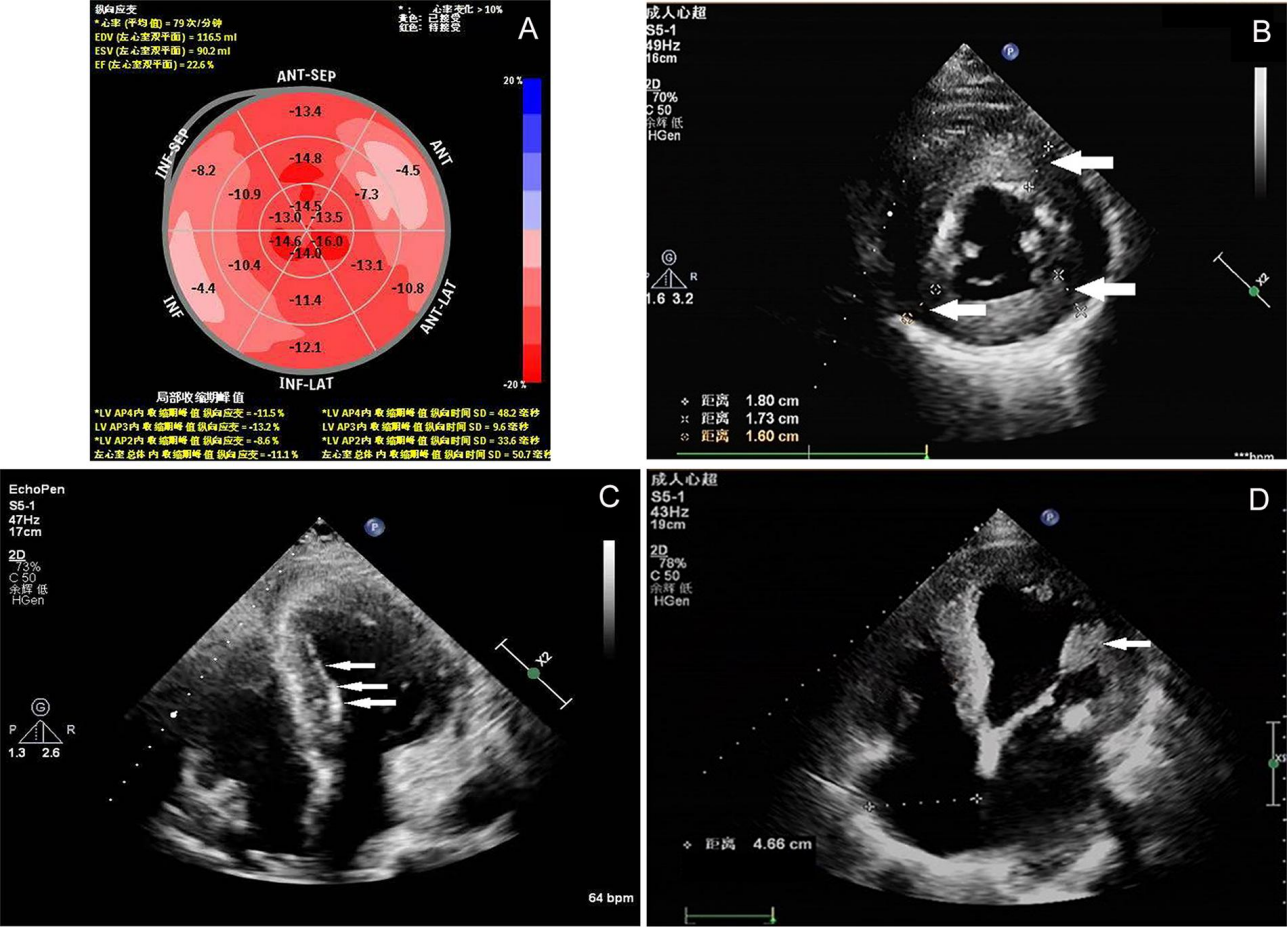
Echocardiographic findings. **(A)** Illustrates reduced global longitudinal strain. **(B)** Demonstrates concentric left ventricular hypertrophy(white arrows). **(C)** Reveals enhanced endocardial echogenicity (binary sign, white arrows). **(D)** Depicts hypertrophy of the anterolateral papillary muscle(white arrows).

Transthoracic echocardiography (Figure 2) identified concentric left ventricular hypertrophy (interventricular septum, 18 mm; posterior wall, 17 mm), endocardial “binary sign”, and enlarged anterolateral papillary muscle. However, it should be noted that the binary sign lacks both sensitivity and specificity for Fabry disease and has been observed in other conditions, including hypertrophic cardiomyopathy (3). Left ventricular ejection fraction was preserved at 60%, while global longitudinal strain was reduced to 11.1%. No regional wall-motion abnormalities were observed.

**Figure 3:**
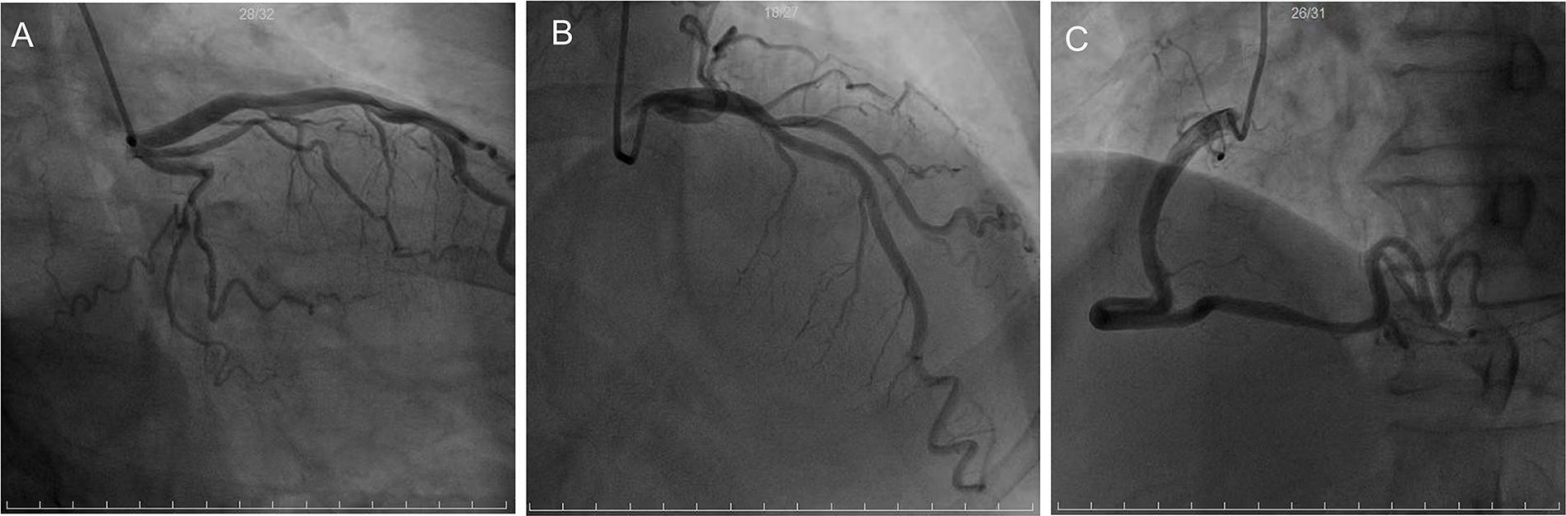
Coronary angiography. Coronary angiography showed unobstructed epicardial vessels with Thrombolysis in Myocardial Infarction (TIMI) grade 3 flow. **(A)** Right anterior oblique caudal view; **(B)** right anterior oblique cranial view; **(C)** left anterior oblique projection.

Coronary angiography (Figure 3) revealed TIMI grade 3 flow in all major coronary arteries without significant stenosis.

Despite clinical and electrocardiographic features mimicking acute ST-segment elevation myocardial infarction, the absence of elevated cardiac biomarkers and obstructive coronary artery disease, combined with echocardiographic findings suggestive of infiltrative cardiomyopathy, prompted targeted evaluation for rare etiologies.

Plasma α-galactosidase A activity was markedly reduced at 0.86 μmol/L/h (reference range, 2.20–17.86), and plasma lyso-Gb3 concentration was elevated at 2.73 ng/mL (reference value, <1.11). Long-range PCR and subsequent Sanger sequencing of the GLA gene identified a hemizygous variant, c.640-801G>A (chrX:100654735).

In the absence of extracardiac manifestations, the patient met diagnostic criteria for cardiac-variant Fabry disease. Due to financial constraints, ERT was deferred. He was managed with symptomatic treatment and lifestyle counseling and discharged. At 2-month follow-up, he remained free of chest pain and syncope.

**Figure 4:**
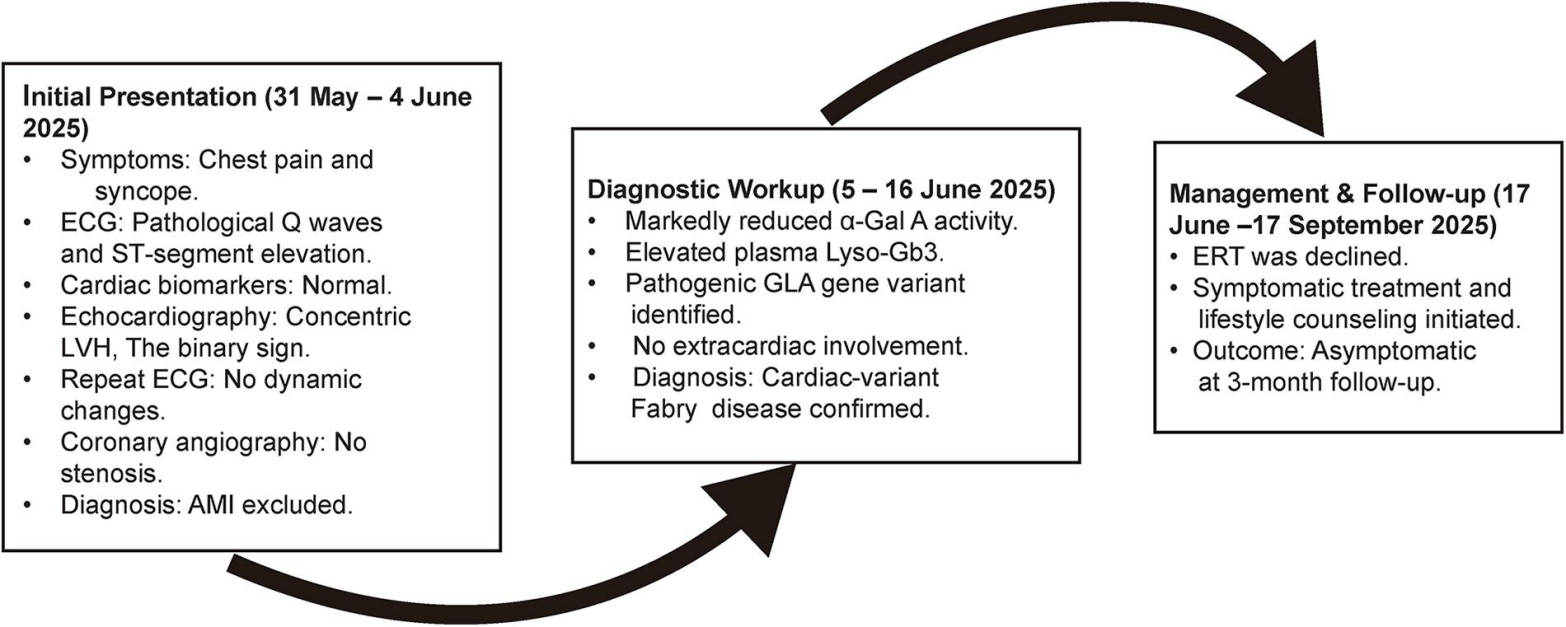
Diagnostic and management timeline. α-Gal A, α-galactosidase A; Lyso-Gb3, globotriaosylsphingosine; LVH, left ventricular hypertrophy; AMI, acute myocardial infarction; ECG, electrocardiogram; ERT, enzyme replacement therapy.

The timeline of the diagnosis and treatment of the patient is shown in Figure 4.

## 3 Discussion

Fabry disease arises from pathogenic variants in the GLA gene located at Xq22.1, which encodes the lysosomal hydrolase α-galactosidase A. Enzyme deficiency leads to progressive lysosomal accumulation of globotriaosylceramide (Gb3) and its deacylated derivative lyso-Gb3 across multiple cell types, resulting in multiorgan dysfunction. Cardiac involvement is the most frequent and remains the leading cause of mortality. Population-based estimates place the prevalence between 1 in 117,000 and 1 in 50,000 live births (4, 5).

The patient presented with angina and syncope; his electrocardiogram demonstrated pathological Q waves and ST-segment elevation in anterior leads, findings that strongly suggest acute myocardial infarction. However, the absence of elevated cardiac biomarkers, lack of dynamic ST-T changes, and angiographically normal coronary arteries excluded acute coronary syndrome. In clinical practice, patients presenting with chest pain and ST-segment elevation without characteristic dynamic changes in myocardial infarction biomarkers require a diagnostic approach that balances the urgent exclusion of obstructive coronary artery disease (CAD) against the inherent risks and benefits of available diagnostic modalities. Coronary computed tomography angiography (CCTA) has recently emerged as a preferred noninvasive anatomical assessment tool for individuals at low to intermediate risk, owing to its high negative predictive value. Multiple clinical trials have demonstrated that an initial CCTA strategy, compared with direct invasive coronary angiography (ICA), significantly reduces procedure-related complications without increasing cardiovascular event rates and facilitates effective patient triage (6). The patient described herein exhibited a clinical presentation consistent with acute coronary syndrome; however, cardiac biomarkers remained persistently negative, and echocardiographic findings were indicative of an infiltrative cardiomyopathy, as evidenced by concentric left ventricular hypertrophy and the binary sign. In this scenario, had CCTA been performed initially and yielded a negative result [e.g., CAD-Reporting and Data System (CAD-RADS) score of 0 or 1], it would have permitted earlier and safer exclusion of ischemic heart disease, thereby enabling prompt diagnostic redirection toward rare cardiomyopathies such as Fabry disease and facilitating earlier initiation of targeted biochemical and genetic evaluations. Although ICA ultimately provided definitive exclusion of CAD—a valid diagnostic approach—this case highlights the evolving role of CCTA in the differential diagnosis of patients presenting with “pseudo-infarction” electrocardiographic patterns. Echocardiography revealed concentric left ventricular hypertrophy, enlarged papillary muscles, and the characteristic binary sign endocardial stripe—key diagnostic features indicative of Fabry disease. This echocardiographic sign reflects regional Gb3 deposition beneath the endocardium, producing a layered, highly reflective interface. The diffusely reduced global longitudinal strain further supports infiltrative fibrosis rather than segmental ischemic dysfunction.

Electrocardiographic manifestations of Fabry disease more commonly include left ventricular high voltage, shortened PR interval, ST-segment depression, T-wave inversion, and conduction abnormalities. In addition to Fabry disease, several other conditions may present with chest pain and ST-segment elevation in the absence of obstructive coronary artery disease. Cardiac amyloidosis, particularly transthyretin amyloid cardiomyopathy (ATTR-CM), can closely mimic Fabry disease by exhibiting left ventricular hypertrophy and pseudo-infarction patterns on electrocardiography, often accompanied by low QRS voltages (7). Acute pericarditis may produce diffuse ST-segment elevation and PR-segment depression, simulating ST-segment elevation myocardial infarction (STEMI) (8). Pulmonary embolism, especially when hemodynamically significant, can manifest as anterior ST-segment changes secondary to right ventricular strain; recent artificial intelligence–based electrocardiogram (AI-ECG) models have proven useful in detecting these patterns (9). Brugada syndrome, although typically characterized by ST-segment elevation in the right precordial leads, may occasionally be misinterpreted as anterior ischemia, particularly in the presence of conduction abnormalities (10). Finally, severe hyperkalemia can cause QRS widening and ST-segment elevation that mimic acute myocardial infarction and may be accompanied by flaccid paralysis (11). These differential diagnoses should be systematically considered and excluded in patients presenting with ischemic-appearing electrocardiograms but unobstructed coronary arteries.

Anterior-lead pseudo-infarction patterns, as observed in this case, are rare and have been reported only sporadically (12), underscoring the disease's phenotypic heterogeneity. Patchy or diffuse myocardial fibrosis replaces viable myocytes with collagen, abolishing local electrical activity and generating pathological Q waves (13). Intralysosomal Gb3 disrupts ion-channel function, prolongs repolarization, and creates transmural voltage gradients that manifest as ST-segment elevation or depression, analogous to a “current of injury” (14). The right bundle branch, owing to its length and superficial location, is particularly susceptible to infiltrative changes; Gb3 accumulation frequently produces conduction delay, rendering right bundle-branch block disproportionately prevalent in cardiac-variant Fabry disease (15). Thus, unexplained left ventricular hypertrophy accompanied by pseudo-infarction electrocardiographic patterns—especially in the absence of biomarker elevation and coronary obstruction—should prompt consideration of Fabry disease.

Beyond epicardial coronary obstruction, coronary microvascular dysfunction (CMD) has been increasingly recognized as a mechanism of myocardial ischemia in Fabry disease. Patients may present with angina, ST-segment abnormalities, or elevated troponin levels despite angiographically normal coronary arteries, a clinical phenotype consistent with ischemia with non-obstructive coronary arteries (INOCA) or myocardial infarction with non-obstructive coronary arteries (MINOCA) (16–18). Graziani et al. demonstrated severe global subendocardial ischemia on positron emission tomography (PET) imaging in patients with advanced Fabry cardiomyopathy, reflecting impaired microvascular vasodilatory reserve (17). Elliott et al. further showed that CMD, assessed by reduced coronary flow reserve, was not reversed by ERT, suggesting that microvascular remodeling may progress independently of substrate clearance (18). These findings underscore the importance of considering CMD in patients with Fabry disease who present with pseudo-infarction electrocardiographic patterns, even in the absence of epicardial coronary stenosis.

Specific therapies include ERT, pharmacologic chaperones, and emerging gene-based approaches. ERT with recombinant α-galactosidase A facilitates catabolism of Gb3 and lyso-Gb3, reducing substrate accumulation and stabilizing organ function. Clinical trials have demonstrated symptomatic improvement and slower progression of organ damage in both early and advanced disease; however, maximal benefit is observed when ERT is initiated prior to irreversible fibrosis (19). Therefore, early diagnosis and timely intervention are imperative.

Limitations of this study include the following: (1) as a single-center case report, the findings require validation in larger-scale studies; (2) the patient's family history was incomplete—although his mother's sudden cardiac death suggested a possible hereditary cardiomyopathy, Fabry disease (FD) could not be definitively confirmed; (3) the patient did not undergo cardiac magnetic resonance imaging (CMR), and the absence of late gadolinium enhancement (LGE) imaging limited further characterization of myocardial involvement, despite CMR's critical role in the differential diagnosis of FD.

## 4 Patient perspective

The patient reported that obtaining a definitive diagnosis of Fabry disease brought clarity following an initial presentation suggestive of myocardial infarction. He acknowledged that ERT was deferred due to financial limitations and concurred with the management strategy of vigilant clinical monitoring. The patient expressed reassurance from regular follow-up visits and maintained a hopeful outlook regarding long-term disease management.

## 5 Conclusion

In patients presenting with pseudo-infarction electrocardiographic changes, normal cardiac biomarkers, angiographically unobstructed coronary arteries, and concentric left ventricular hypertrophy in the absence of volume or pressure overload, Fabry disease should be included in the differential diagnosis. Echocardiographic findings—such as papillary muscle hypertrophy, the characteristic binary sign, and globally reduced longitudinal strain—should raise clinical suspicion. Definitive diagnosis relies on the triad of leukocyte *α*-galactosidase A activity measurement, plasma lyso-Gb3 quantification, and GLA gene sequencing; the latter remains the diagnostic gold standard and facilitates cascade family screening and early, disease-modifying therapy.

## Data Availability

The datasets presented in this article are not readily available due to privacy/ethical restrictions. Requests to access the datasets should be directed to the corresponding author.
